# Supplementary material for: A self-regulating shuttle for autonomous seek and destroy of microplastics from wastewater
Source: Nat Commun. 2025 Jul 21;16:6707. doi: 10.1038/s41467-025-61899-4 (PMC12279941; doi:10.1038/s41467-025-61899-4)
Supplement: Supplementary file 1 — Supplementary Information [file 41467_2025_61899_MOESM1_ESM.pdf]

A Self-Regulating Shuttle for Autonomous Seek and Destroy of Microplastics from Wastewater

Dennis Kollofrath[a], Florian Kuhlmann[a], Sebastian Requardt[a], Yaşar Krysiak[a] and Sebastian Polarz[a]*

a) Department of Inorganic Chemistry, Leibniz-University of Hannover, 30167 Hannover, Germany

Supplementary Information

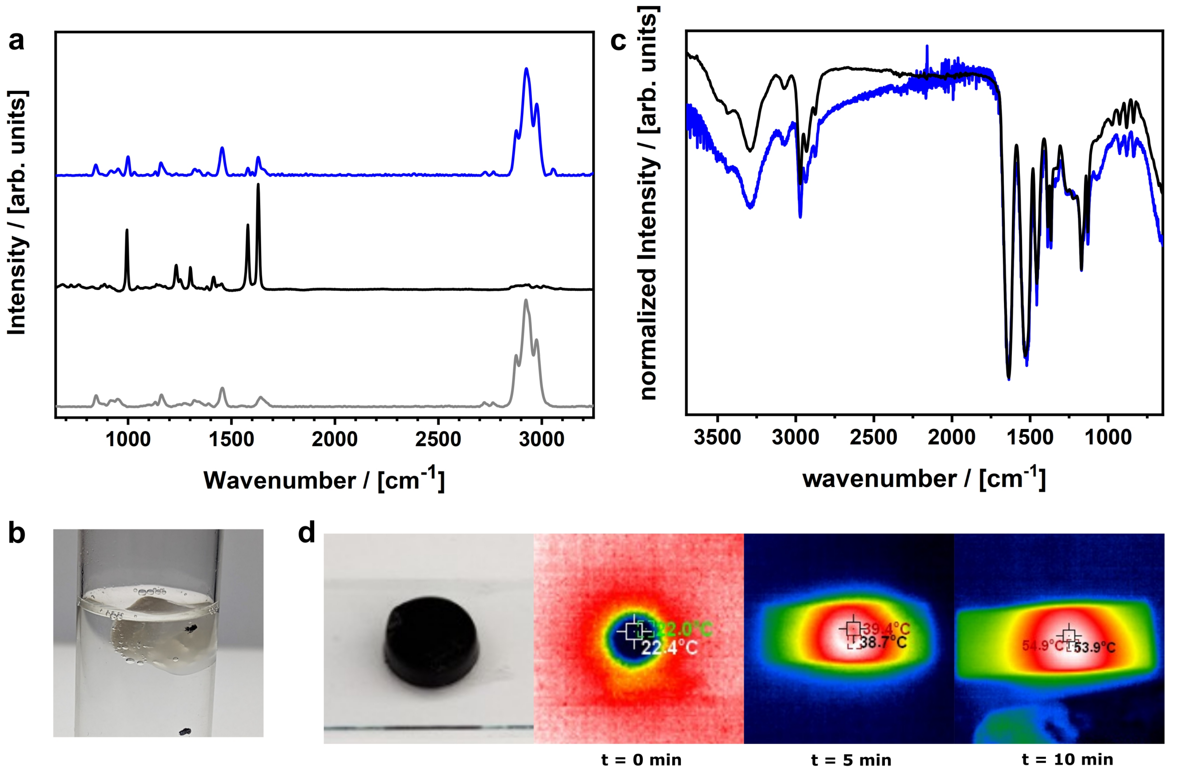

Figure S1. Preparation and characterization of the BDS-gel including polydopamine coating. **a** Raman spectrum of the hybrid hydrogel shuttle (blue), the NOPs (black) and pNIPAM (grey). **b** Photographical image of the resulting hybrid hydrogel shuttle. **c** Infrared spectra before (black) and after (blue) coating of the BDS-gel with polydopamine. **d** Photothermal heating of the BDS-gel under a solar simulator after different time intervals.

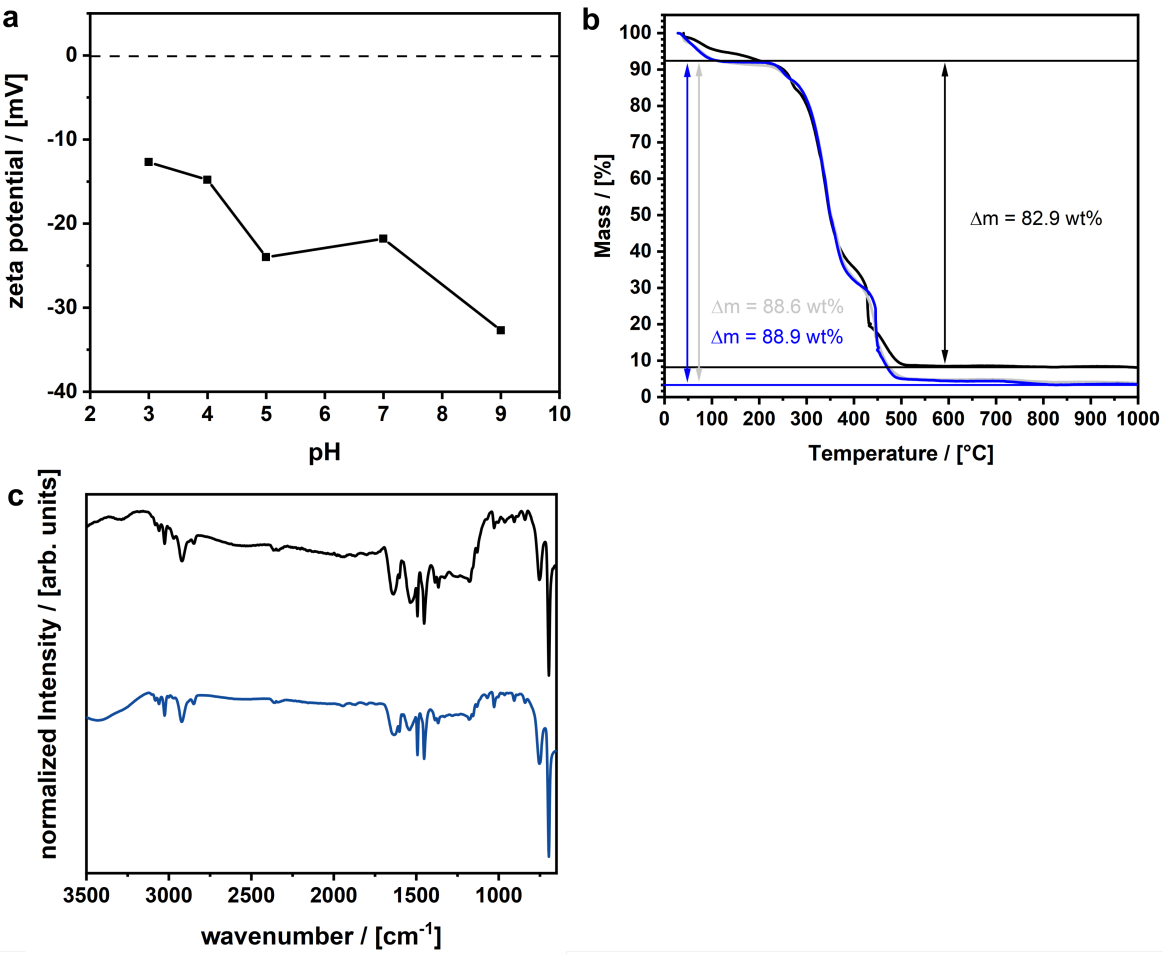

Figure S2. Additional analytics on the microplastic uptake by the BDS-gel. **a** pH-dependent zeta potential of the polystyrene latex beads used as microplastic reference. **b** Thermogravimetric analysis of BDS-gels before (black) and after (grey) microplastic uptake (2 µm polystyrene beads) and after washing (blue). A single washing cycle involves heating the gel to 40°C in salt water (0.1 wt%) for 3 hours to induce collapse, followed by immersion in fresh water at 20°C for 6 hours to allow swelling. This cycle is repeated three times.**c** IR-spectra of a BDS-gel treated with a polystyrene solution before (black) and after (blue) washing.

Calculations from TGA

82.9 wt% weight loss before microplastic uptake
88.6 wt% weight loss after microplastic uptake

Δm = 5.7 wt% (microplastics content) => 57 mg per gramm of adsorbent

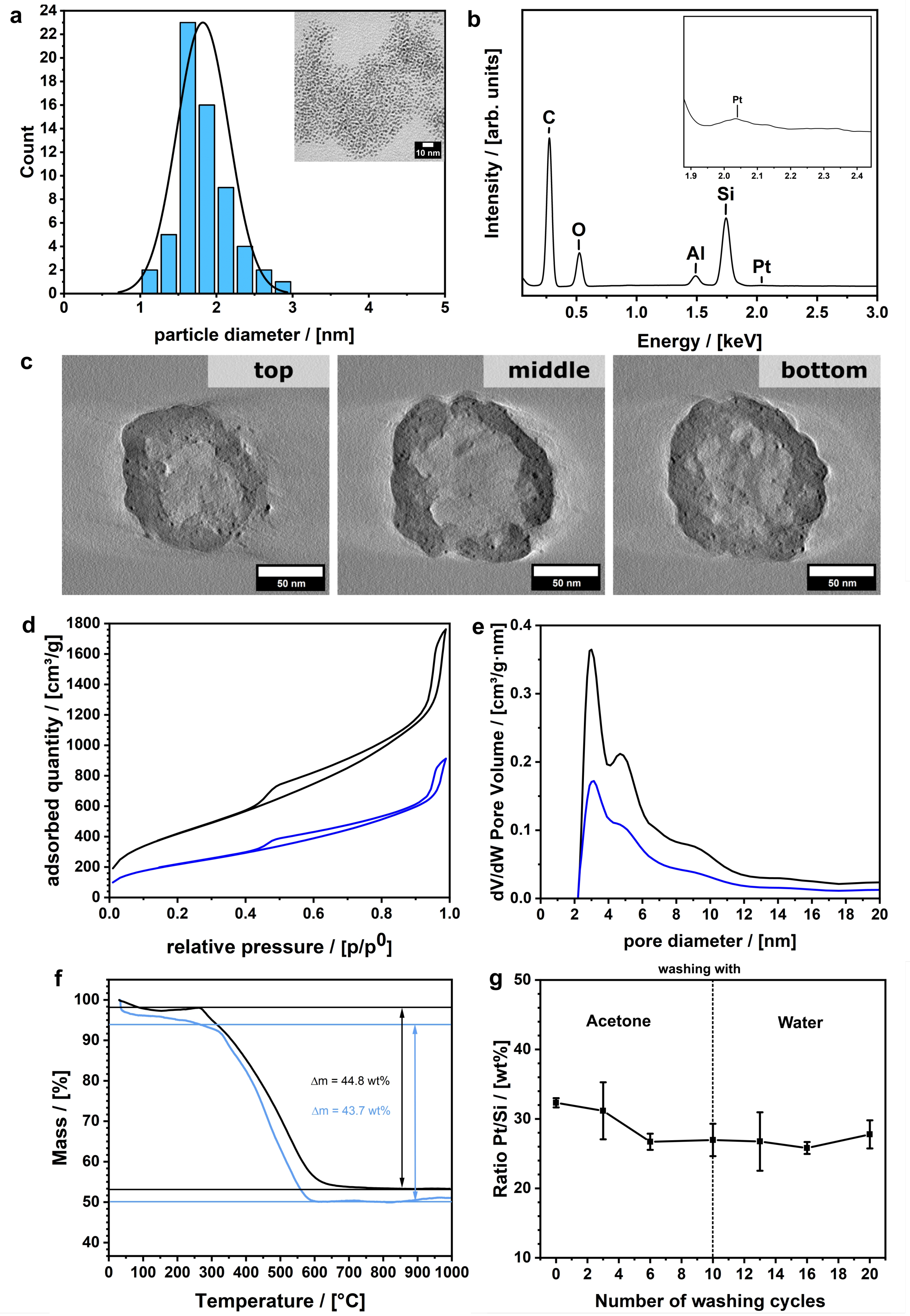

Figure S3. Functionalization of the inner surface of the NOPs with platinum nanoparticles. **a** Particle size distribution of the Pt nanoparticles (Pt-NPs) introduced into the pore system of the NOPs, derived from the TEM micrograph shown in the inset (scale bar = 10 nm). **b** EDX spectrum of the NOPs after wet-impregnation with Pt-NPs. **c** Three representative slices from a

TEM tomogram of Pt-NP-functionalized NOPs, corresponding to the top, middle, and bottom regions of the NOPs. **d** N_2-Physisorption isotherms of the NOPs before (black) and after (blue) wet-impregnation with Pt-NPs. **e** NLDFT Pore size distribution of the NOPs before (black) and after (blue) wet-impregnation with Pt-NPs**. f** Thermogravimetric analysis of the NOPs before (black) and after (blue) wet-impregnation with Pt-NPs. **g** Si/Pt ratio in the functionalized NOPs after ten washing cycles each with acetone and water, as determined by EDX measurements. Error bars represent the maximum range observed across three to five individual measurements each

Calculation from TGA

- From the reference (black): 44.8 wt% of the unfunctionalized NOPs are burned during TGA
- 55.2 wt% remain as SiO_2
- Sample weight: 5.5 mg
- 44.8 wt% of 5.5 mg → 2.47 mg
- 55.2 wt% of 5.5 mg → 3.06 mg should remain
- From experiment (blue)
- 43.7 wt% of the functionalized NOPs are burned during TGA (2.40 mg)
- 56.3 wt% remain (3.10 mg)
- 0.9 wt% (0.06 mg) of the sample is platinum

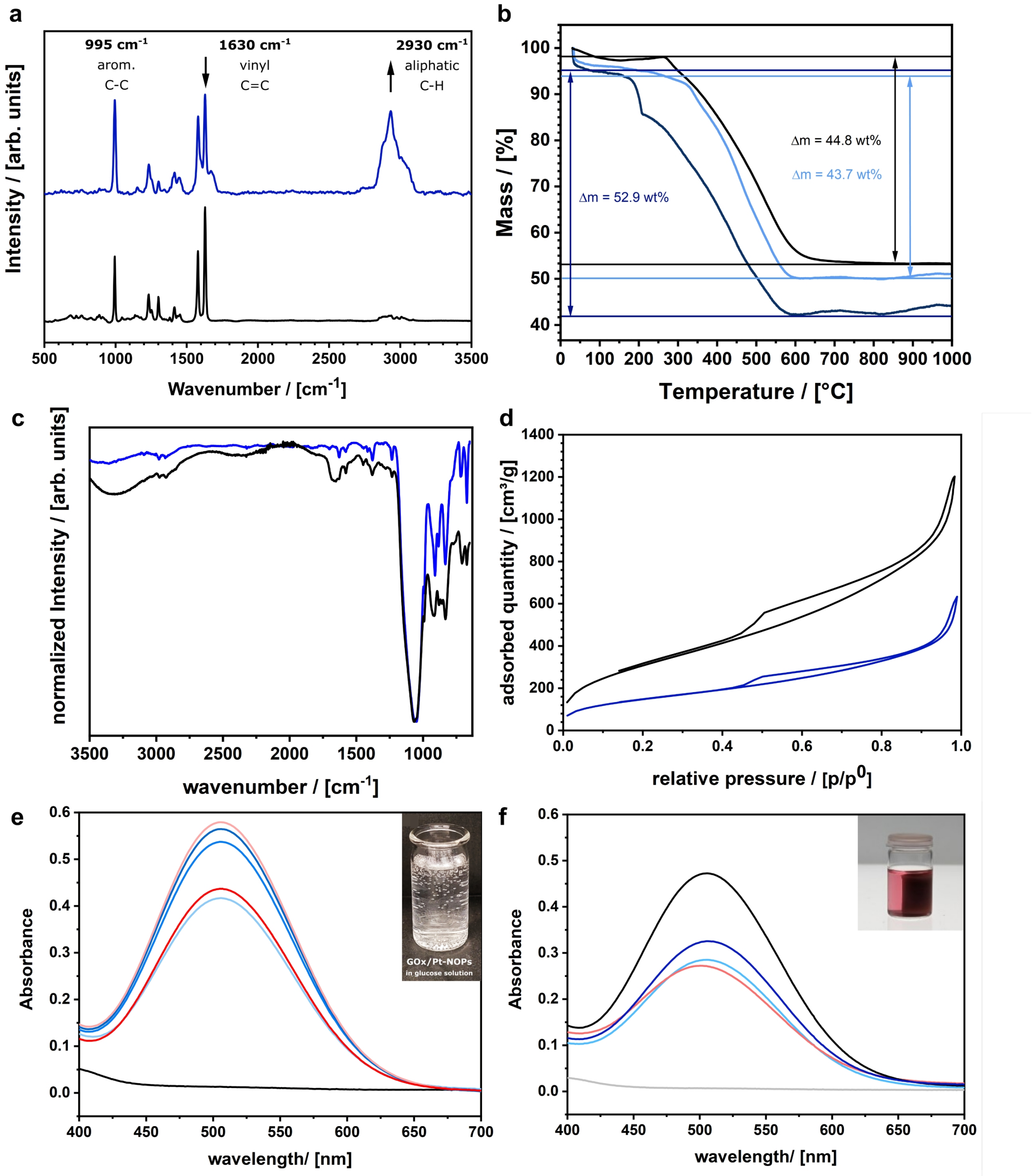

Figure S4. Immobilization of glucose oxidase on NOPs and its enzymatic activity. **a** Raman spectrum of the NOPs before (black) and after (blue) the functionalization with Glucose Oxidase. **b** Thermogravimetric analysis of the unfunctionalized NOPs (black), after the wet-impregnation with Pt-NPs (light blue) and after thiol-ene-click reaction with glucose oxidase (Gox) (dark blue). **c** Infrared spectra of the NOPs before (black) and after (blue) the functionalization with GOx. **d** N_2-Physisorption isotherms of the NOPs before (black) and after (blue) the functionalization with GOx. **e** UV/Vis spectra of the GOx activity assay using GOx-functionalized NOPs at different temperatures (raw data corresponding to Fig. 2b). The black curve represents the negative control (no GOx). Spectra were recorded at 10 °C (light blue), 20 °C (blue), 30 °C (dark blue), 40 °C (light red), and 50 °C (red).Inset demonstrates oxygen formation of GOx/Pt-NOPs in glucose solution. **f** UV/Vis spectra of the GOx activity assay using BDS-gels containing GOx-functionalized NOPs at different temperatures. The grey curve represents the negative control (no GOx), the black curve represents the positive control (pure

GOx). Spectra were recorded at 10 °C (dark blue), 20 °C (light blue) and 40 °C (red). Inset shows image of the resulting BDS-gel after the glucose oxidase assay.

Calculations from the TGA:

from NOPs to Pt-NOPs

44.8 wt% weight loss (unfunctionalized NOPS)
43.7 wt% weight loss (Pt-NOPS)
Δm = 0.9 wt% => 0.9 wt% Pt content

From Pt-NOPs to GOx/Pt-NOPs

43.7 wt% weight loss (Pt-NOP)
52.9 wt% weight loss (GOx/Pt-NOP)
Δm = 9.2 wt% => 9.2 wt% GOx on the NOPs

Calculations from the UV/Vis study of the BDS-gel (S4f):

Temperature [°C]	Enzyme activity [Units/g]
10	6.26
20	5.40
40	5.04

a

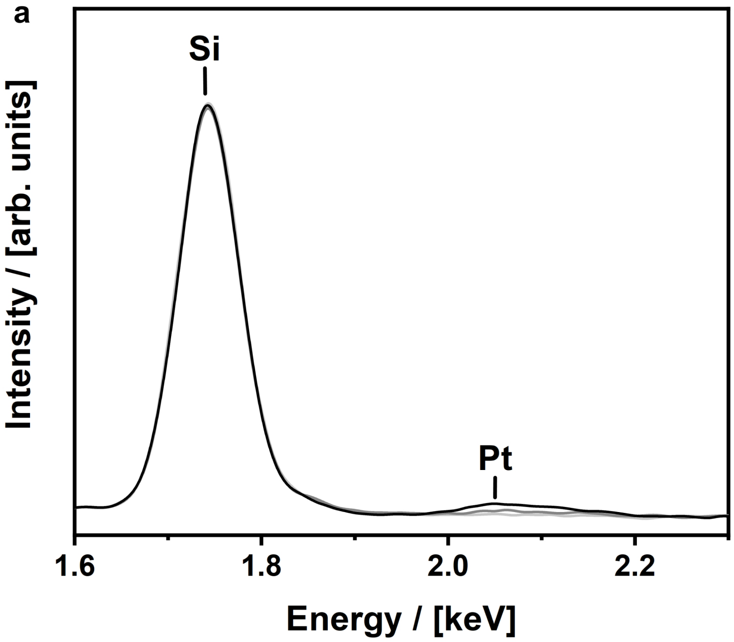

b

Sample	weight percentages [wt%]	atomic percentages [at%]
black	1.6	0.13
dark grey	0.8	0.07
light grey	0.2	0.02

c

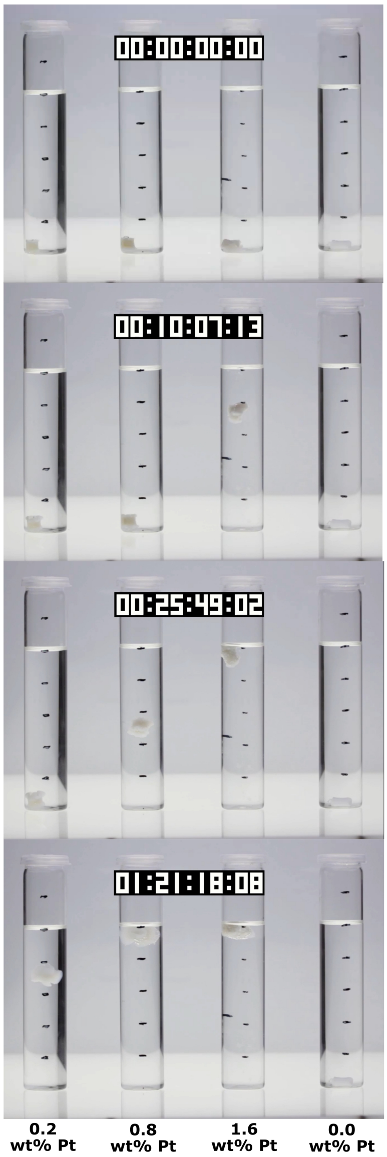

Figure S5. Effect of catalyst amount variation on the ascent process**. a** EDX measurements of NOPs containing different Pt-NP loadings (0.2 wt%, 0.8 wt%, and 1.6 wt%). **b** atomic percentages of Pt in the NOPs derived from the EDX measurements**. c** Movie analysis of the ascent behavior of BDS-gels prepared from NOPs with varying platinum contents, recorded at different time intervals (for full movie, see Movie S1). Three batches of NOPs were prepared, each with a different platinum content (0.2 wt%, 0.8 wt%, and 1.6 wt%). These nanoparticles were then converted into BDS-gels. The gels were immersed in a 0.3% hydrogen peroxide solution, and the time was measured until the gels began to ascend. For simplicity, glucose oxidase was intentionally excluded from this experiment, as its presence does not affect the overall trend. The results indicate that the ascent time of the gels can be controlled by adjusting the catalyst concentration, with an inverse proportionality observed between the two variables.

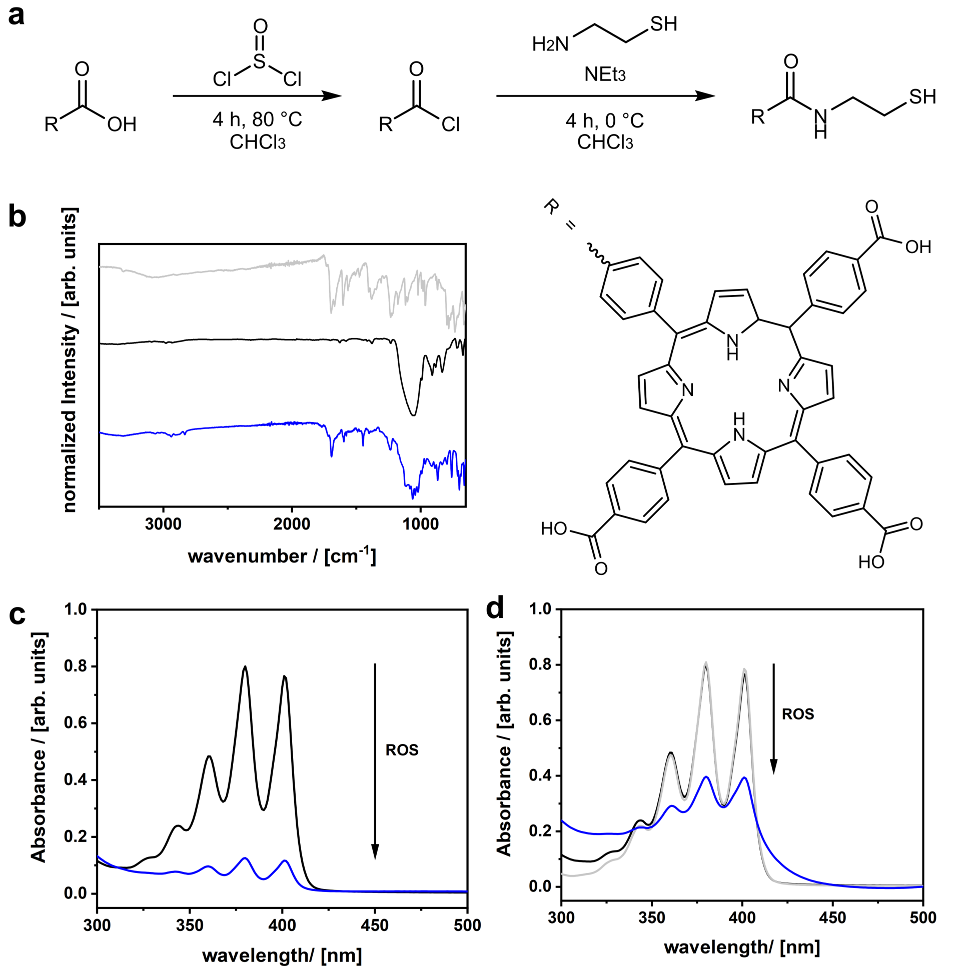

Figure S6. Functionalization of NOPs with the photosensitizer and their ROS-generating properties. **a** Reaction path for the thiol modification of tetrakis-(4-carboxyphenyl) porphyrin (TCPP) to make it suitable for thiol-ene click reaction with the NOPs. **b** IR spectra of the TCPP functionalized NOPs before (black) and after (blue) functionalization and pure TCPP (grey) as reference. **c** ABDA (9,10-Anthracenediyl-bis(methylene)dimalonic acid) assay monitoring singlet oxygen production by TCPP-functionalized NOPs. UV/Vis spectra show the initial ABDA concentration at t = 0 min (black) and after 120 min treatment with TCPP-functionalized NOPs (blue), demonstrating ABDA degradation due to singlet oxygen generation. **d** ABDA assay of BDS-gels containing TCPP-functionalized NOPs. UV/Vis spectra show the initial ABDA concentration at t = 0 min (black) and the decreased ABDA concentration after 120 min treatment (blue). An ABDA assay of a BDS-gel without the photosensitizer was also performed (grey). The absence of ABDA degradation in the non-functionalized gel confirms that singlet oxygen generation is due to the photosensitizer and not to ABDA adsorption by the gel matrix.

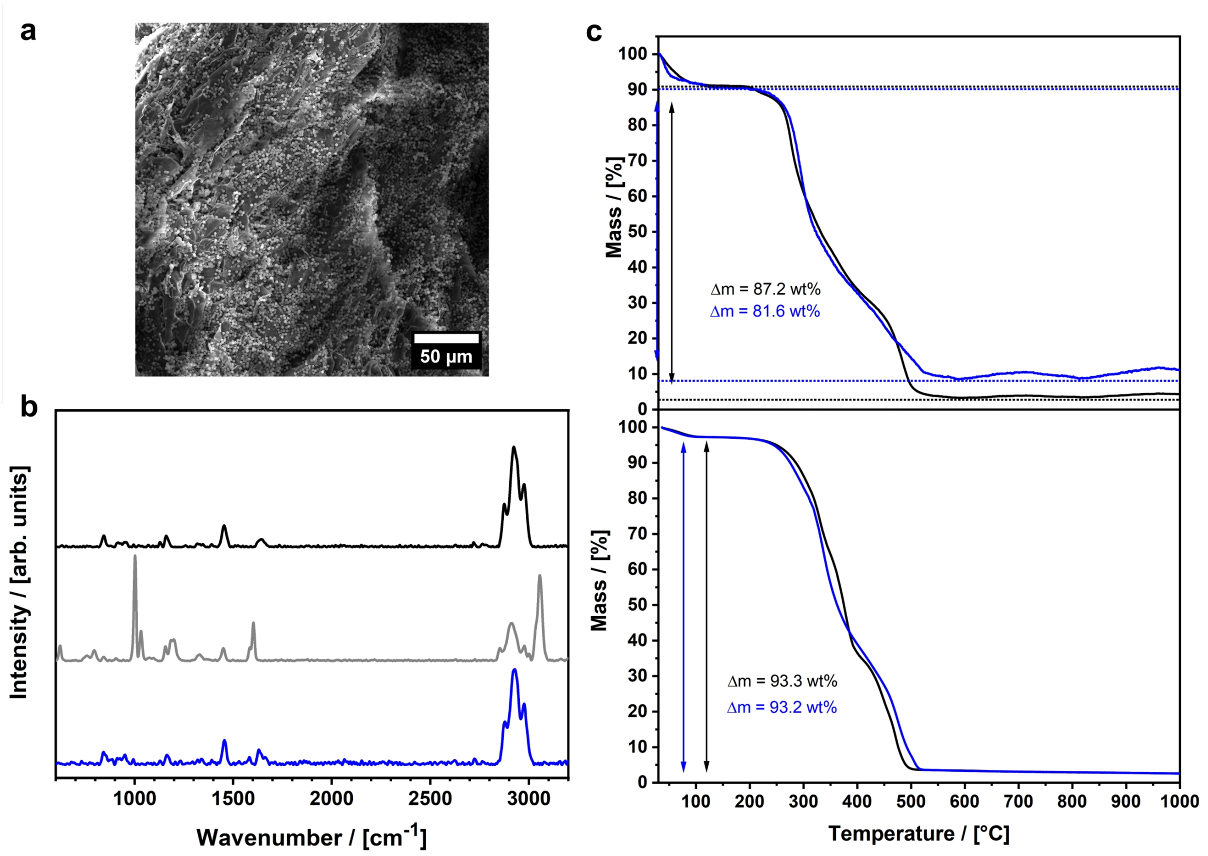

Figure S7. Microplastic degradation performance of the BDS gel. **a** SEM micrograph of the microplastic loaded BDS-gel without the photosensitizer after irradiation. **b** Raman spectra of the BDS-gel before (black) and after (grey) loading with polystyrene beads and after irradiation with UV light (blue). **c** Thermogravimetric analysis of microplastic loaded BDS-gels before (black) and after (blue) the irradiation. Two different BDS-gels were used: Top image: BDS-gel with photosensitizer on the NOPs, bottom image: BDS-gel without photosensitizer on the NOPs.

<u>Calculations from TGA:</u>

Amount of microplastic on the BDS-gels 5.7 wt%

with photosensitizer (MP Uptake 5.7 wt%]:
87.2 wt% - 81.6 wt% = 5.6 wt%
5.6 wt% / 5.7 wt% = 0.982 → 98.2 % of polystyrene was removed

without photosensitizer (MP uptake: 2.4 wt%):
93.3 wt% - 93.2 wt% = 0.1 wt%
0.1 wt% / 2.4 wt% = 0.041 → 4.1 % of polystyrene was removed

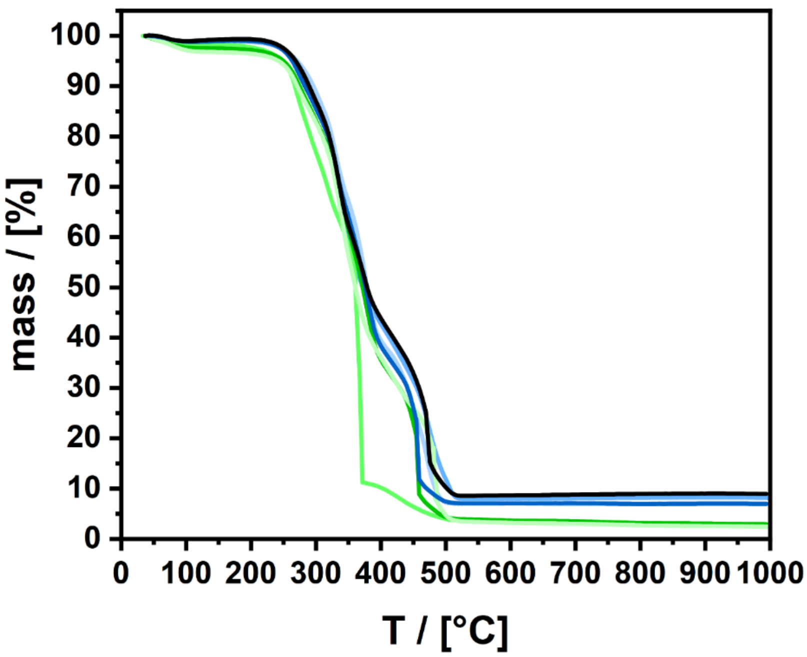

Figure S8. Efficiency of microplastic degradation over multiple cycles determined from thermogravimetric analysis of BDS-gels.

Table 1: Microplastic loading and decomposition determined from TGA measurements.

Cycle	Phase	m_{start} [wt%]	m_{end} [wt%]	m_{diff} [wt%]	$m_{MP\ before\ irradiation}$ a) [wt%]	$m_{MP\ after\ irradiation}$ b) [wt%]	Removal Efficieny c) [%]
1	before Loading	99.4	8.4	91.0			
	after Loading	97.4	2.4	95.0	4.0	0	100.0
	after irradiation	99.1	8.1	91.0			
2	before Loading	99.1	8.1	91.0			
	after Loading	97.5	2.9	94.6	3.6	0.1	97.2
	after irradiation	98.1	7.0	91.1			
3	before Loading	98.1	7.0	91.1			
	after Loading	98.4	2.6	95.8	4.7	0.4	91.5
	after irradiation	98.6	7.1	91.5			

From cycle 2 onwards, the value for m (microplastic after irradiation) corresponds to the value of m (microplastic before loading). (a) the amount of microplastic loaded on the BDS-gel is calculated by subtraction of m_{diff} before loading with m_{diff} after loading; (b) the amount of microplastic loaded on the BDS-gel after irradiation is calculated by subtraction of m_{diff} after irradiation with m_{diff} before loading; (c) Removal efficiency is calculated by dividing the amount of MP after and before irradiation.

Table 2: Comparison of the MP content leaving the solution and MP in the BDS-gel.

Cylce	$MP_{leaving\ solution}$ [mg]	$MP_{gel\ (TGA)}$ [mg/g]	$m_{gel;\ used}$ [mg}	$M_{Gel\ (calculated)}$ [mg]	Difference [mg]
**1**	**5.2**	40	100	**4.0**	+1.2
**2**	**3.4**	36	80	**2.9**	+0.5
**3**	**3.2**	47	60	**2.8**	+0.4

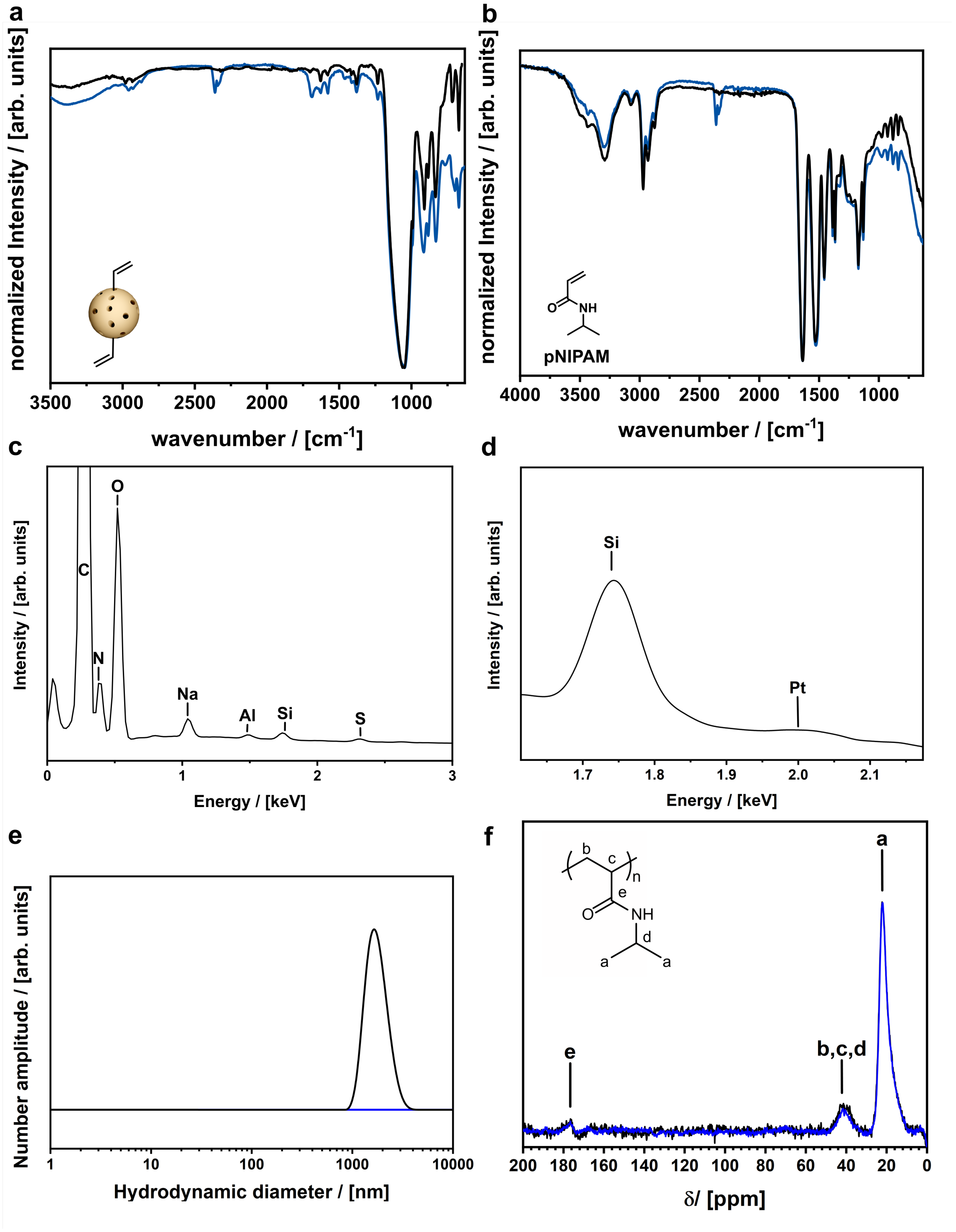

Figure S9. Assessment of ROS stability of the BDS gel and further microplastic degradation analyses. **a** IR spectra of NOPs before (black) and after (blue) ROS treatment. **b** IR spectra of pNIPAM before (black) and after (blue) ROS treatment. **c** EDX-spectrum of the BDS-gel after irradiation with a sunlight simulator. **d** Zoomed-in view of the EDX-spectrum in (c) highlighting the platinum signal. **e** Dynamic light scattering measurement of the washing solution after microplastic decomposition (blue). A DLS measurement of the initial polystyrene dispersion (2 µm beads) was also performed as a reference (black). No smaller fragments or microplastic beads were detected indicating that the decomposition was either quantitative or down to small organic molecules. **f** high-power decoupling with magic angle spinning (HPDec-MAS) NMR of the BDS-Gel before (balck) and after (blue) the irradiation with a sunlight simulator. MAS solid state NMR is too insensitive to show NOPs or polystyrene in the material.

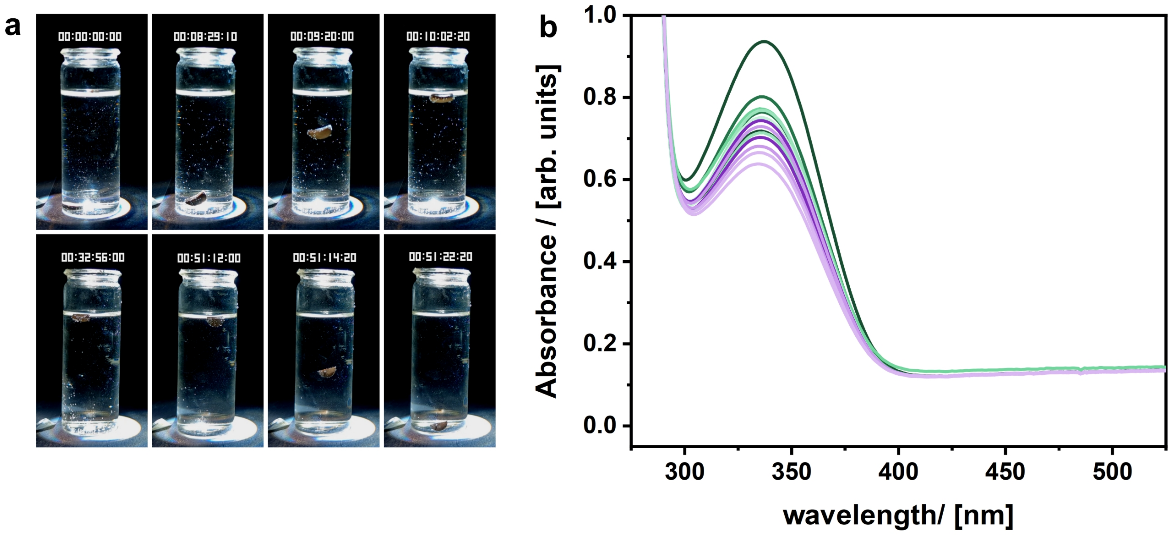

Figure S10. Descending process of the BDS-gel **a** Key frames from the movie analysis of the ascending and descending behavior of the BDS-gel under sunlight irradiation, illustrating one complete cycle. (For the full movie, see Movie S2.). **b** Time-resolved D-glucose concentration studied by UV-Vis assay (raw data to Figure 4f). A glucose concentration assay was performed after different time intervals. From dark to light green: t = 0, 5 ,10, 15, 20, 30, 45, 60, 75, 90 minutes. From dark to light purple: t = 105, 120, 135, 150, 165, 180 minutes.

Figure S11. Estimated operational/material costs for a BDS-gel

**Material**	**Costs for one Hydrogel [€]**
NIPAM	0.52
Dopamine	1.41
$Na_2PtCl_4 \cdot 3H_2O$	0.24
Glucose Oxidase	2.57
Porphyrin	4.60
NOPs (starting from the basic materials)	15.00
Other chemicals	1,00
Solvents	7.50
Energy	30
**Total** (for 110 mg BDS-gel)	**65.34**
**1 g BDS-gel**	**588.06**
**without energy**	**318.0**

Since the BDS hydrogel system operates autonomously, the ongoing operational costs are minimal. The system relies on natural resources, such as sunlight and glucose, which are freely available and do not incur additional costs
