# Supplementary material for: A self-regulating shuttle for autonomous seek and destroy of microplastics from wastewater
Source: Nat Commun. 2025 Jul 21;16:6707. doi: 10.1038/s41467-025-61899-4 (PMC12279941; doi:10.1038/s41467-025-61899-4)
Supplement: Supplementary file 2 — Description of Additional Supplementary Files [file 41467_2025_61899_MOESM2_ESM.pdf]

Description of Additional Supplementary Files

File name: Supplementary Movie 1
Description: Catalyst-dependent buoyancy activation of hybrid hydrogels in hydrogen peroxide. Supplementary Movie S1 shows four BDS-gels immersed in a 0.3% H_2O_2 solution, each containing a different platinum content (0 wt%, 0.2 wt%, 0.8 wt%, and 1.6 wt%) embedded in the nanoporous organosilica nanoparticles (NOPs). The gels with increasing platinum loading exhibit faster oxygen formation, leading to earlier onset of buoyancy and vertical ascent. The hydrogel without platinum remains at the bottom, confirming the catalytic origin of oxygen generation. The embedded timer indicates the time required for each sample to ascend.

File name: Supplementary Movie 2
Description: Autonomous buoyancy cycle of the BDS-gel under irradiation by a solar simulator. Supplementary Movie S2 demonstrates a full buoyancy cycle of the BDS-gel in a glucose solution. Initially, the gel swells and ascends due to oxygen formation. Upon reaching the surface, exposure to solar irradiation induces heating, causing the thermoresponsive matrix to collapse and expel the gas, leading to a controlled descent. The timer overlay tracks the duration of the full up-and-down cycle.
